# Supplementary material for: To treat or not to treat: Assessing the role of anti-enterococcal therapy for intra-abdominal infections in patients with cancer
Source: PLoS One. 2024 Feb 7;19(2):e0298018. doi: 10.1371/journal.pone.0298018 (PMC10849250; doi:10.1371/journal.pone.0298018)
Supplement: Attachment — Submitted filename: Review. docx.docx [file pone.0298018.s002.docx]

**Review**

- **Abstract – Line 46-47 :** Retrospective analysis shouldn’t provide recommendations to change practice. It should provide recommendation to test the outcome in a prospective randomised trial to confirm the results.
- **In exclusion criteria , Line 109-114** – Exclusion due to treatment with levofloxacin /meropenem doesn’t feel justified given their marginal/negligible activity against Enterococci. These patients might be receiving those drugs to target other organism concurrently awaiting culture reports. Even after culture reports, if they still didn’t receive appropriate treatment, they can simply be put in “no appropriate treatment” arm.
- **Table 2** – Univariate and multivariate analysis – the factor “multiple micro-organisms” should also be tested on univariate analysis. This might be relevant as 1) it might indicate more severe sepsis 2). It was unequally distributed in both arms.

Rather than taking Pitt bacteremia score cut-off of 2, different cut-offs can be tested in univariate analysis to see if value above a certain cut-off is associated with increased mortality.

- **Line 242-246** : The word “ outcome” isn’t clear. What exactly the authors mean by the term “outcome”.
- As there were only 3 patients with hematologic malignancies (which represents a totally different profile of immunocompromised patients compared to solid cancers), to make the study more homogenous and generalizable , it would be better to include only solid cancer patients. As such 3 patients is too small a sample size for representing hematological malignancies in the study.
- **Conclusion** : Its not advisable to recommend a practice change just based on one retrospective study which doesn’t rule out any inherent bias due to retrospective nature of study design, unless a good quality systematic review of all pooled retrospective data also draw same conclusion. Here, the conclusion should suggest a prospective randomised trial to confirm the observed findings.
