# Supplementary material for: To treat or not to treat: Assessing the role of anti-enterococcal therapy for intra-abdominal infections in patients with cancer
Source: PLoS One. 2024 Feb 7;19(2):e0298018. doi: 10.1371/journal.pone.0298018 (PMC10849250; doi:10.1371/journal.pone.0298018)
Supplement: Attachment — Submitted filename: Response-to-editor.docx [file pone.0298018.s003.docx]

**RESPONSE TO EDI COMMENTS**

**Our point-by-point responses to the comments and suggestions by the Editor are listed below.**

Dear Editor:

Thank you for your constructive comments. We have carefully revised our manuscript as suggested, and your insightful comments have helped us significantly improve the quality of our manuscript. The page and line numbers in the responses refer to the relevant parts of the main manuscript (changes marked in red font) where the text has been revised.

**Editor:**

**Comment 1:**

Use the term 'ascitic fluid' consistently throughout the manuscript.

**Response 1:** Thank you for your valuable suggestions. We have corrected this.

Page 2, line 32：

ascitic fluid cultures.

Page 4, line 60:

Enterococci are found in 20%–30% of ascitic fluid cultures

Page 6, line 104：

intraoperative ascitic fluid cultures.

Page 10, line 161：

Ascitic fluid samples were stained using Gram stain and cultured

Page 12, line 203：

in which enterococci were detected in ascitic fluid cultures (Fig 1).

Figure1

**Comment 2:**

Line 62: Ascetic fluid- Please correct the typological error.

**Response 2:** Thank you for pointing this out. We have corrected this.

Page 4, lines 62–63：

Moreover, intra-abdominal infections, in which enterococci are isolated from ascitic fluid,

**Comment 3:**

Please justify the objective which mentions "empirical administration of agents effective against enterococci". It is important because antimicrobial susceptibility testing was carried out and targeted treatment was given against enterococci at least in one group of patients i.e., "appropriate treatment group". Thereby the anti-enterococcal treatment given to patients cannot exactly be an empiric treatment.

**Response 3:** Thank you for your very constructive remarks. By "effective empiric therapy for enterococci," I meant "treatment including enterococci as a target was initiated before the ascitic fluid culture was identified, and the patient was treated with that antimicrobial for at least 4 days." We have added clarification where appropriate.

Page 4, lines 67–69：

Consequently, there are no definitive guidelines regarding which patients should receive empiric therapy with antimicrobial agents targeting enterococci before culture results are known.

Page 5, lines 82–85：

Therefore, this study aimed to determine whether the empirical administration of agents effective against enterococci before culture results are known is crucial for the treatment of intra-abdominal infections in immunocompromised patients with cancer.

Page 7, lines 120–123：

"The initiation of therapy that includes enterococci as a target before ascites cultures are known and continues for at least 4 days" was defined as "effective empiric therapy for enterococci." Therefore, we first divided the patients into two groups: those who received treatment covering enterococci and those who did not.

Page 7-8, lines 126–128：

Patients who did not receive treatment covering enterococci were defined as those who did not meet the above definition.

Page 8, lines 129–130：

if one type of enterococci was present and the treatment did not cover that enterococci, the patient was classified in the "untreated patients" group.

**Comment 4:**

Table 1: The annotations mentioned in row 1 for Age is not clear. What do the statistical figures 66.1 and 69.9 refer to? What statistical method has been adopted to reach to the p value for comparison of age in treated versus untreated groups? Please clarify.

**Response 4:** Thank you for your valuable suggestions. 66.1 and 69.9 are means. P-values were obtained using Student's t-test. The notation was not clear, so it was changed. The value of standard deviation was also added.

Page 13, Table1

**Comment 5:**

It will be better to mention the groups as "patients with appropriate anti-enterococcal treatment" and "patients without appropriate anti-enterococcal treatment" rather than "Treated patients" and "Untreated patients".

**Response 5:** Thank you for pointing this out. We have corrected it.

Page 2, lines 35–36：

 In total, 103 patients were included: 61 received treatment covering enterococci, and 42 did not.

Page 7, lines 123–126：

“Treated patients” was defined as initiating treatment in patients with antimicrobial agents that exhibit susceptibility toward the enterococci detected in ascites fluid within 48 h of acquiring ascites.

Page 12, lines 203–204：

Of these, 61 patients received treatment covering enterococci, whereas 42 did not.

Page 13, lines 230–231：

Table 1. Comparison of the clinical characteristics of the patients with and without treatment covering enterococci

Figure1

**Comment 6:**

As per Figure 1, if the same Enterococcus species was detected on the same day, it was counted as one case. Does it mean that if same Enterococcus species was detected in same patient on subsequent days, that patient was considered as more than one case? If latter is the true, authors need to relook into the entire statistical calculation. Please clarify.

**Response 6:** Thank you for your valuable comments.

If the same enterococci species was detected in the same patient the next day, it was counted as one case. No such case occurred because patients with a history of antimicrobial therapy were excluded. I indicate the exclusion criteria in the text.

Pages 6–7, lines 107–109

The exclusion criteria were age <18 years, intra-abdominal infection without microbiological culture, no intra-abdominal infection (e.g., routine drain culture after surgery), patient on antimicrobials at the time of culture collection,

**Comment 7:**

As per Materials and methods, patients receiving levofloxacin and meropenem were excluded from the study. However, the figure 1 mentions only meropenem treatment as one of the criteria for exclusion.

**Response 7:** Thank you for pointing this out. Since no patient received levofloxacin, only meropenem was listed in Figure 1. We would like to add to the text.

Page 7, line 117：No patients in this study received levofloxacin.
