# Supplementary material for: To treat or not to treat: Assessing the role of anti-enterococcal therapy for intra-abdominal infections in patients with cancer
Source: PLoS One. 2024 Feb 7;19(2):e0298018. doi: 10.1371/journal.pone.0298018 (PMC10849250; doi:10.1371/journal.pone.0298018)
Supplement: Attachment — Submitted filename: Response to Reviewers.docx [file pone.0298018.s004.docx]

**RESPONSE TO THE REVIEWER’S COMMENT**

**Our response to the comment by Reviewer＃1 is provided below.**

Dear Reviewer:

Thank you for your constructive comments. We have carefully revised our manuscript as suggested, and your insightful comment has helped us significantly improve the quality of our manuscript. The page and line numbers in the below response refer to the relevant parts of the main manuscript where the text has been revised (the changes are marked in red font).

**Reviewer #1:**

**Comment 1:**
Thanks for considering revision of the manuscript after the comments. However, the point of empiric versus definitive therapy is still not addressed very convincingly.

**Response 1:** Thank you for highlighting this issue. We have changed the expression "empiric therapy" to "initial antimicrobial therapy" and made the corresponding changes to the relevant parts of the manuscript as indicated below. In addition, we have included an additional section of text providing further information, which is also provided below.

Page 2, lines 27–30:

In this study, we evaluated the impact of the initial antimicrobial therapy administration of anti-enterococcal agents on the treatment of intra-abdominal infections in patients with cancer in whom enterococci were isolated from ascitic fluid cultures.

Page 3, lines 46–48:

The results of this study suggest that the initial routine administration of anti-enterococcal agents for intra-abdominal infections may not be essential for all patients with cancer.

Page 5, lines 83–86:

Therefore, this study aimed to determine whether the initial administration of agents effective against enterococci before culture results are known is crucial for the treatment of intra-abdominal infections in immunocompromised patients with cancer.

Pages 7–8, lines 121–130:

Patients were defined as "receiving effective initial therapy for enterococci" if antimicrobial therapy was initiated within 48 hours of ascitic fluid collection, the enterococci detected in the ascitic fluid were susceptible to that antimicrobial therapy, and treatment was continued for at least 4 days. Patients were divided into two groups, with the group that received the above-defined effective initial therapy against enterococci classified as "treated patients" and the group that did not receive treatment defined as "untreated patients”. In cases where multiple types of enterococci were detected in the ascitic fluid, if one or more types of enterococci were present that were not covered by appropriate treatment, the patient was classified as part of the "untreated patients" group.

Pages 20–21, lines 291–293:

Therefore, based on the results of these studies, we propose the empiric administration of anti-enterococcal agents from the beginning only for intra-abdominal infections in severely ill patients with cancer.

Page 23, lines 341–343:

Our findings suggest that not all cancer patients require initial antimicrobial therapy against enterococci and that treatment should be considered for severe cases and patients with PS2 or higher.
